# First isolation of a novel tottorivirus from wild boar faeces in Japan

**DOI:** 10.1099/jgv.0.002334

**Published:** 2026-10-07

**Authors:** Mami Oba, Ai Hirabayashi, Natsuko Teshima, Hitoshi Takemae, Toru Oi, Fujiko Fukuda, Shoichi Sakaguchi, Takanori Shiga, Naoyuki Aihara, Junichi Kamiie, Hiroho Ishida, Hironobu Murakami, Tetsuya Mizutani, Takeshi Noda, Makoto Nagai

**Affiliations:** 1 Center for infectious Disease Epidemiology and Prevention Research, Tokyo University of Agriculture and Technology, Fuchu, Tokyo 183-8509, Japan; 2 One Health Infectious Disease Control Research Hub, Tokyo University of Agriculture and Technology, Koganei, Tokyo 184-0012, Japan; 3 Research Unit of One Health Infectious Disease Control, Advanced Research Center for One Welfare, Tokyo University of Agriculture and Technology, Koganei, Tokyo 184-0012, Japan; 4 School of Veterinary Medicine, Azabu University, Sagamihara, Kanagawa 252-5201, Japan; 5 Laboratory of Ultrastructural Virology, Institute for Life and Medical Sciences, Kyoto University, Kyoto 606-8507, Japan; 6 Faculty of Bioresources and Environmental Science, Ishikawa Prefectural University, Nonoichi, Ishikawa, 921-8836, Japan; 7 Ishikawa Nanbu Livestock Hygiene Service Center, Kanazawa, Ishikawa 920–3101, Japan; 8 Department of Microbiology and Infection Control, Faculty of Medicine, Osaka Medical and Pharmaceutical University, Takatsuki, Osaka 569-8686, Japan; 9 Laboratory of Ultrastructural Virology, Graduate School of Biostudies, Kyoto University, Kyoto 606-8507, Japan; 10 Institute for Integrated Cell-Material Sciences, Kyoto University, Kyoto 606-8507, Japan

**Keywords:** cell culture, genetic diversity, recombination, tottorivirus, tropism, wild boar

## Abstract

Viruses of the genus *Tottorivirus* (family *Picornaviridae*) have been identified in pigs but no isolates have been reported to date. In this study, a novel tottorivirus strain, designated Tottori/tc/Wild Boar/Japan/Ishi-6/2020 (Ishi-6), was isolated for the first time from a faecal sample of a wild boar using swine testis cells, where cytopathic effects (CPEs) were observed. Ultrathin-section transmission electron microscopy revealed virus-like structures arranged around vesicular membranes in infected cells. The Ishi-6 replicated in Porcine Kidney-15 and Cloned Porcine Kidney cells with CPEs but no replication was observed in non-porcine cell lines. Sequence analysis showed that Ishi-6 shared high aa sequence identity with a U.S. pig slurry-derived strain in the polyprotein, especially 2C+3 CD region with the highest identity (95.16%). In contrast, the P1 region was more closely related to a Chinese pig-derived strain (74.18%), while showing low identity to Japanese pig-derived tottorivirus strains. Phylogenetic analysis divided tottoriviruses into three clusters based on the polyprotein sequence, whereas different clustering patterns were observed for the P1 and 2C+3 CD regions. The incongruent clustering of Ishi-6 between these regions suggested recombination, which was further supported by recombination analysis identifying a breakpoint near the VP1–2A junction, involving U.S. pig slurry-derived and Chinese pig-derived strains. Real-time reverse transcription PCR targeting the 5′ UTR of Ishi-6 was performed on faecal samples from 280 wild boars and 70 domestic pigs collected between 2018 and 2023. Nine wild boar samples collected between 2018 and 2022 tested positive, while no positive sample was obtained from pigs. These findings indicate that Ishi-6 is a putative recombinant tottorivirus that may have circulated in Japanese wild boar populations. Tottoriviruses exhibit considerable genetic diversity, likely driven by recombination, and continued surveillance is warranted, including their pathogenicity in pigs.

## Data availability

The nucleotide sequences of Tottori/wt/Pig/Japan/Uchi-12/2019 and Tottori/tc/Wild Boar/Japan/Ishi-6/2020 have been deposited in GenBank/EMBL/DDBJ/PIR under the accession number LC900407 and LC934724.

## Introduction

Viruses of the family *Picornaviridae* are small, icosahedral, non-enveloped viruses with highly diverse positive-sense, ssRNA genomes. They are ubiquitous and globally distributed, and several members pose significant threats to human and livestock health [1, 2]. The family *Picornaviridae* currently comprises 159 species assigned to 68 genera (as of March 2024), which are classified into five subfamilies, *Caphthovirinae*, *Kodimesavirinae*, *Ensavirinae*, *Paavivirinae* and *Heptrevirinae*, based on phylogenetic analyses of the 3 CD (virus-encoded proteinase and polymerase precursor) and P1 (capsid protein precursor) coding regions [3]. The genus *Tottorivirus* belongs to the subfamily *Caphthovirinae* together with the genera *Ailurivirus*, *Aphthovirus*, *Bopivirus*, *Cardiovirus*, *Cosavirus*, *Erbovirus*, *Hunnivirus*, *Malagasivirus*, *Marsupivirus*, *Mischivirus*, *Mosavirus*, *Mupivirus*, *Senecavirus*, *Teschovirus* and *Torchivirus*. Members of this subfamily are characterized by cleavage of the capsid precursor 1AB (VP0) into 1A (VP4) and 1B (VP2), as well as by the presence of a leader protein [3].

The prototype strain of tottorivirus (TotV), Tottori/wt/Pig/WOL/Japan/2015 (WOL), was first identified in a faecal sample from a healthy 2-month-old pig in Tottori Prefecture, Japan. Subsequently, TotVs were detected in 23.3% of faecal samples collected from pigs younger than 4 months of age in Japan by reverse transcription PCR (RT-PCR) targeting the 3D region [4]. However, no studies have investigated the presence of TotVs in wild boars in Japan. To date, only a single species has been assigned to this genus, and merely 11 near-complete and three partial genome sequences have been reported from the United States, China, Vietnam and South Africa [5–7] (as of May 2026). Furthermore, no successful isolation of TotVs has been reported, and their biological properties remain largely unknown.

During enteric virome surveillance of Japanese wild boars, we identified a novel TotV strain, designated Tottori/tc/Wild Boar/Ishi-6/Japan/2020 (Ishi-6), following inoculation of swine testis (ST) cells with a faecal sample from a wild boar. In this study, we aimed to characterize this virus by electron microscopy, assessment of cell tropism, genome analysis and comparative genomic analyses with previously reported TotV sequences available in the GenBank/EMBL/DDBJ/PIR database.

## Methods

### Isolation and propagation of Ishi-6

During enteric virome research using juvenile wild boars, Ishi-6 was isolated from a 13 kg female wild boar with no clinical symptoms captured on 28 July 2020, in Ishikawa Prefecture, Japan. Virus isolation using ST cells has been described previously [8]. Briefly, the faecal supernatant was activated with an equal volume of 5 µg/ml L-1-tosylamide-2-phenylmethyl chloromethyl ketone (TPCK)-trypsin (Worthington) in Eagle’s minimal essential medium (E-MEM, Nissui) for 1 h at 37 °C and inoculated into ST cells. Confluent ST cells in 24-well plates were washed twice and infected with 0.1 ml of the activated sample, followed by 60 min adsorption at 37 °C. After washing with E-MEM three times, cells were cultured in E-MEM containing 0.3% BSA (Fujifilm Wako Pure Chemical) and 0.5 µg ml^−1^ TPCK-trypsin at 37 °C with 5% CO_2_ for 7 days. If no cytopathic effect (CPE) appeared, samples were freeze–thawed three times and passaged similarly. Seed virus was propagated using the ST cells by passaging.

### Sample preparation for ultrathin-section transmission electron microscopy

ST cells infected with Ishi-6 were fixed with 2.5% glutaraldehyde in cacodylate buffer and postfixed with 1% osmium tetroxide on ice. Samples were then dehydrated through ethanol gradients, substituted with propylene oxide and embedded in epoxy resin. Ultrathin sections (~50 nm) were prepared using an ultramicrotome (EM UC7; Leica, Wetzlar, Germany). The sections were stained with uranyl acetate and lead citrate and observed with an HT-7700 TEM (transmission electron microscopy) (Hitachi High-Tech Corporation, Japan) at 80 kV.

### Cells and virus growth kinetics

ST, Porcine Kidney-15 cells (PK-15), Cloned Porcine Kidney cells (CPK), MA104 African green monkey fetal kidney cells (MA104), MARC-145 African green monkey kidney epithelial cells derived from MA104 (MARC-145), Vero simian kidney epithelial cells (Vero), human embryonic kidney 293 cells expressing the SV40 large T antigen (293T), HRT-18 human rectal tumour-derived cells (HRT-18), Baby Hamster Syrian Kidney-21 cells (BHK-21), Felis catus whole foetus-4 cells (Fcwf-4), Madin–Darby Canine Kidney cells (MDCK) and Madin–Darby Bovine Kidney cells (MDBK) were maintained in Dulbecco’s modified Eagle’s medium (Fujifilm Wako Pure Chemical) supplemented with 10% FBS at 37 °C.

Ishi-6 at fourth viral passage was used for comparing viral replication efficiency. Since this virus exhibits weak CPEs, making it difficult to detect CPE in 96-well plates, the m.o.i. was defined based on the number of viral genome copies measured by real-time RT-PCR real-time reverse transcription PCR (RT-qPCR). Each cell was seeded in six-well plates and infected with Ishi-6 at ~100 genomic copies per cell. After an adsorption period of 1 h at 37 °C, the inoculum was removed and the cells were washed three times with PBS to eliminate unadsorbed viral particles. E-MEM containing 0.5 µg ml^−1^ TPCK-trypsin was then added to each well, which was designated as time point 0. Cell culture supernatants were harvested at 4 days post-infection (dpi). Total RNA was extracted from the supernatant using NucleoSpin^®^ RNA Virus (Takara Bio), and viral RNA copies were quantified using RT-qPCR. RT-qPCR was performed on a LightCycler^®^ 96 System (Roche) using the One Step PrimeScript™ RT-PCR Kit (Perfect Real Time) (Takara Bio) with a primer–probe set targeting the 5′ UTR (forward primer: 5′-AGGGAAAGCTCGTCCGTACT-3′, reverse: 5′-GCCATGTTAACGTGCCTGTG-3′ and probe: 5′-56-FAM/ACCTCCCCT/ZEN/CACAACATGGCT/3IABkFQ-3′). All experiments were performed in triplicate to ensure reproducibility.

### RNA extraction, cDNA library construction, deep sequencing and determination of 5′ and 3′ terminal nt sequences

Total RNA was extracted from the supernatants of ST cells infected with Ishi-6 using TRIzol LS Reagent (Life Technologies). A cDNA library was constructed for deep sequencing using the NEBNext Ultra II RNA Library Prep Kit for Illumina (New England Biolabs), according to the manufacturer’s instructions. After quantification using a Qubit^®^ 4.0 Fluorometer (Invitrogen), libraries were subjected to paired-end sequencing (2×151 nt) on a MiniSeq benchtop sequencer (Illumina).

Raw sequencing data were processed using Local Run Manager Ver 4.2.0 (Illumina) to generate FASTQ-formatted files. The FASTQ data were imported into CLC Genomics Workbench 25.0.3 (CLC bio), where reads were trimmed and low-quality sequences were removed. The processed reads were assembled *de novo* into contigs using the default settings in CLC Genomics Workbench. The resulting contigs were analysed using the blast programme on the National Center for Biotechnology Information website to identify TotV-related sequences.

The 5′-terminal nt sequence was determined using DNA linker-ligated 5′ rapid amplification of cDNA ends (RACE) using 5′-Full RACE Core Set (Takara Bio) with gene-specific primers. The 3′-terminal sequence was amplified by RT-PCR using gene-specific forward and a reverse oligo primer [9] (Table S1, available in the online Supplementary Material).

### Genome analysis

The genome sequence of Ishi-6 was aligned with those of TotVs and related viruses within the *Caphthovirinae* retrieved from the GenBank/EMBL/DDBJ/PIR databases using clustalW implemented in mega12 [10]. Phylogenetic analyses were performed based on the aa sequences of each genomic region using the maximum-likelihood (ML) method in mega12, with the best-fit substitution models applied (LG+G for polyprotein and 2C+3 CD and LG+G+F for P1). The robustness of the phylogenetic trees was evaluated by bootstrap analysis with 1,000 replicates [11]. Similarity and recombination analyses were conducted using SimPlot v.3.5.151 and RDP (Recombination Detection Program) v.5.552, respectively [12, 13]. Pairwise sequence identities were calculated for each genomic region using the CLC Genomics Workbench. The 5′ UTR and 3′ UTR RNA secondary structure was predicted using RNAfold in the ViennaRNA package (version 2.4.18) [14].

### LDA-based host prediction

The Linear Discriminant Analysis (LDA) model was trained using 276 reference positive-sense ssRNA (+ssRNA) viruses with known host classifications. The resulting model was then applied to classify viral genomes and visualize clustering patterns according to their predicted host associations [15, 16].

### Prevalence investigation of Ishi-6 by RT-qPCR

Rectal contents from 280 juvenile wild boars (~<6 months of age) with no clinical symptoms, hunted in the central region of the main island of Japan (Ishikawa and Toyama Prefectures) between 2018 and 2023 (35 in 2018, 52 in 2019, 23 in 2020, 34 in 2021, 66 in 2022 and 70 in 2023), were used for the prevalence investigation. In addition, faecal samples were directly collected from the rectums of 70 weaned pigs from 7 farms in Ishikawa Prefecture in 2021. All samples were diluted 1 : 9 (wt/v) in E-MEM and centrifuged at 12,000 ***g*** for 10 min. The supernatants were subjected to RNA extraction using magLEAD 6gC (Precision System Science Co., Ltd). RT-qPCR was performed on a LightCycler^®^ 96 System using the One Step PrimeScript™ III RT-qPCR Mix (Takara Bio) with the same primer–probe set used for virus growth kinetics. Samples with cycle threshold (Ct) values of <35 were considered positive.

## Results

### Isolation of Ishi-6 using ST cells

ST cells inoculated with a faecal suspension containing Ishi-6 exhibited CPE after two blind passages. Following infection with Ishi-6, the cells gradually lost their spindle shape and underwent pronounced rounding, and the cell monolayer was partially disrupted at 3 dpi. These rounded cells began to detach from the plastic surface, resulting in a net-like disruption of the monolayer (Fig. 1a, b).

**Fig. 1.**
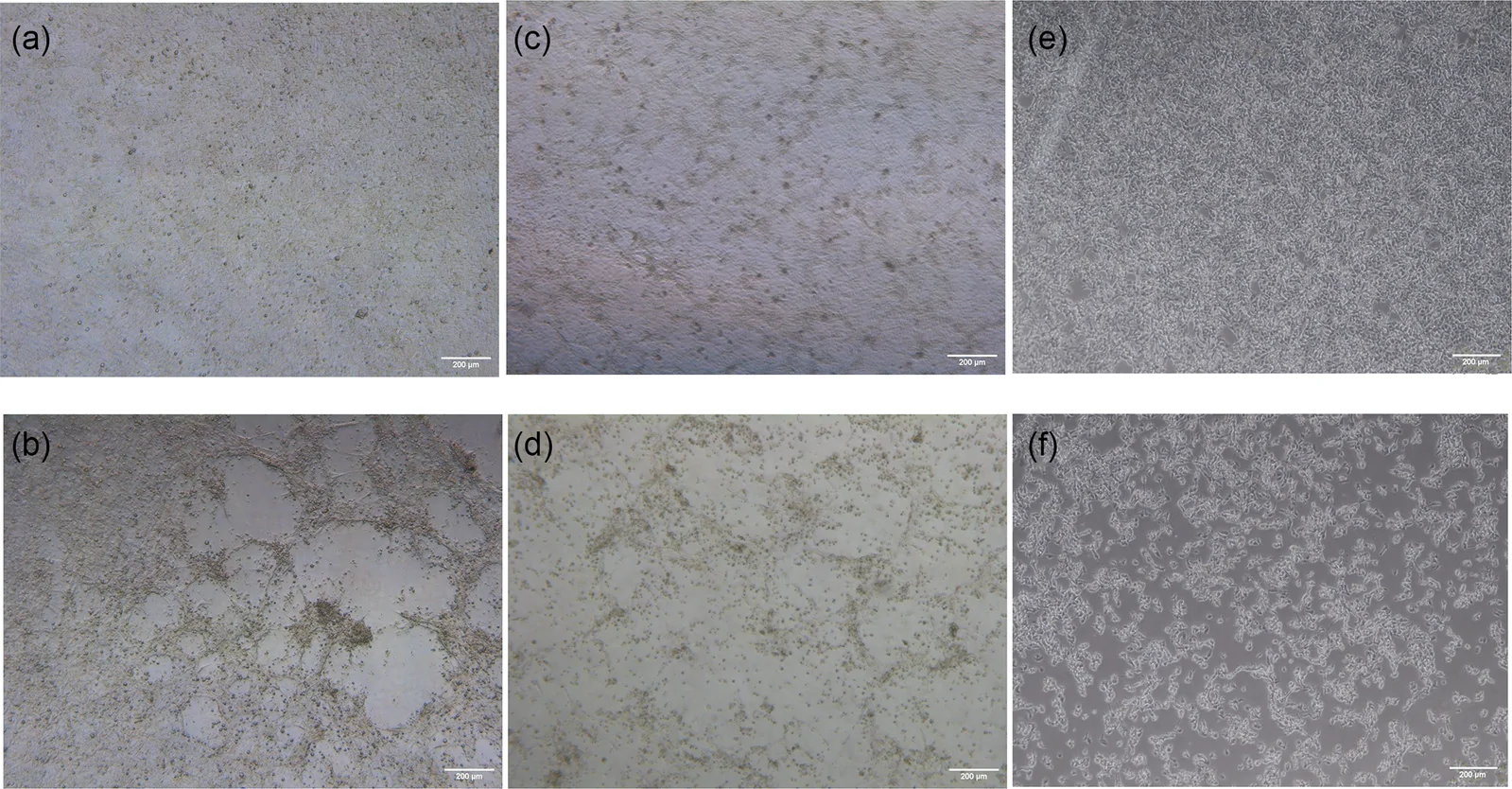
CPE caused by Tottori/tc/Wild Boar/Japan/Ishi-6/2020. Uninfected ST cells (**a**), PK15 cells (**c**) and CPK cells (**e**) observed 72 h after infection. CPE in infected ST cells (**b**), PK15 cells (**d**) and CPK cells (**f**) morphology on 72 h.

### Electron microscopy of Ishi-6 infected ST cells

Ultrathin-section TEM of Ishi-6-infected ST cells at 2 dpi revealed virus-like structures ~30 nm in diameter around vesicles in the cytoplasm, forming a quasicrystalline array (Fig. 2). These structures were not observed in uninfected cells, suggesting that they represent TotV particles.

**Fig. 2.**
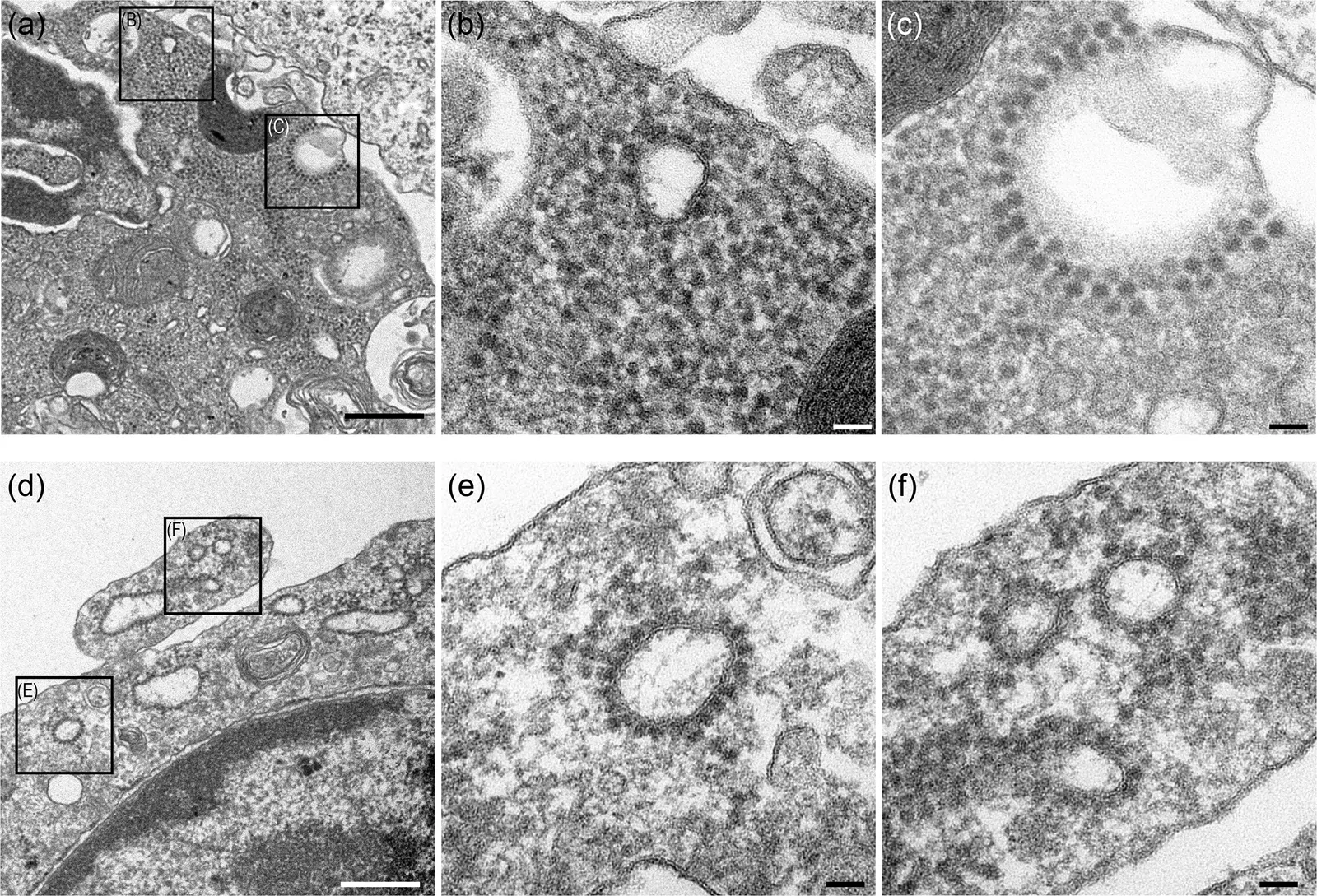
Electron micrographs of Ishi-6-infected ST cells. Virus-like structures arranged around vesicular membranes were observed in Ishi-6-infected cells. Scale bars: (**a**) 50 nm, (**b**) 500 nm, (**c**) 50 nm, (**d**) 50 nm, (**e**) 500 nm, and (**f**) 50 nm.

### Virus growth in cell cultures

The virus growth in ST, CPK, PK-15, MA104, MARC145, Vero, 293T, HRT-18, BHK-21, Fcwf-4, MDCK and MDBK cells was examined using Ishi-6 at four viral passages at ~100 genomic copies per cell. ST, PK15 and CPK cells showed CPE on 3 dpi (Fig. 1c–f), while Ishi-6 inoculated MA104, MARC145, Vero, 293T, HRT-18, BHK-21, Fcwf-4, MDCK and MDBK cells did not exhibit CPE. However, the Ishi-6 did not exhibit strong CPE against ST, PK15 or CPK; RT-qPCR results were positive even in wells from dilution series that showed no CPE, and no plaques formed in any of the cells (data not shown). Therefore, virus growth was examined by RT-qPCR targeted 5′ UTR. The viral genome titre was 2.35×10^7^, 2.34×10^7^ and 2.05×10^6^ copies 0.1 ml^−1^ in 4 dpi supernatant of ST, PK15 and CPK cells, respectively, and no virus growth in other cells was confirmed by RT-qPCR (Fig. 3).

**Fig. 3.**
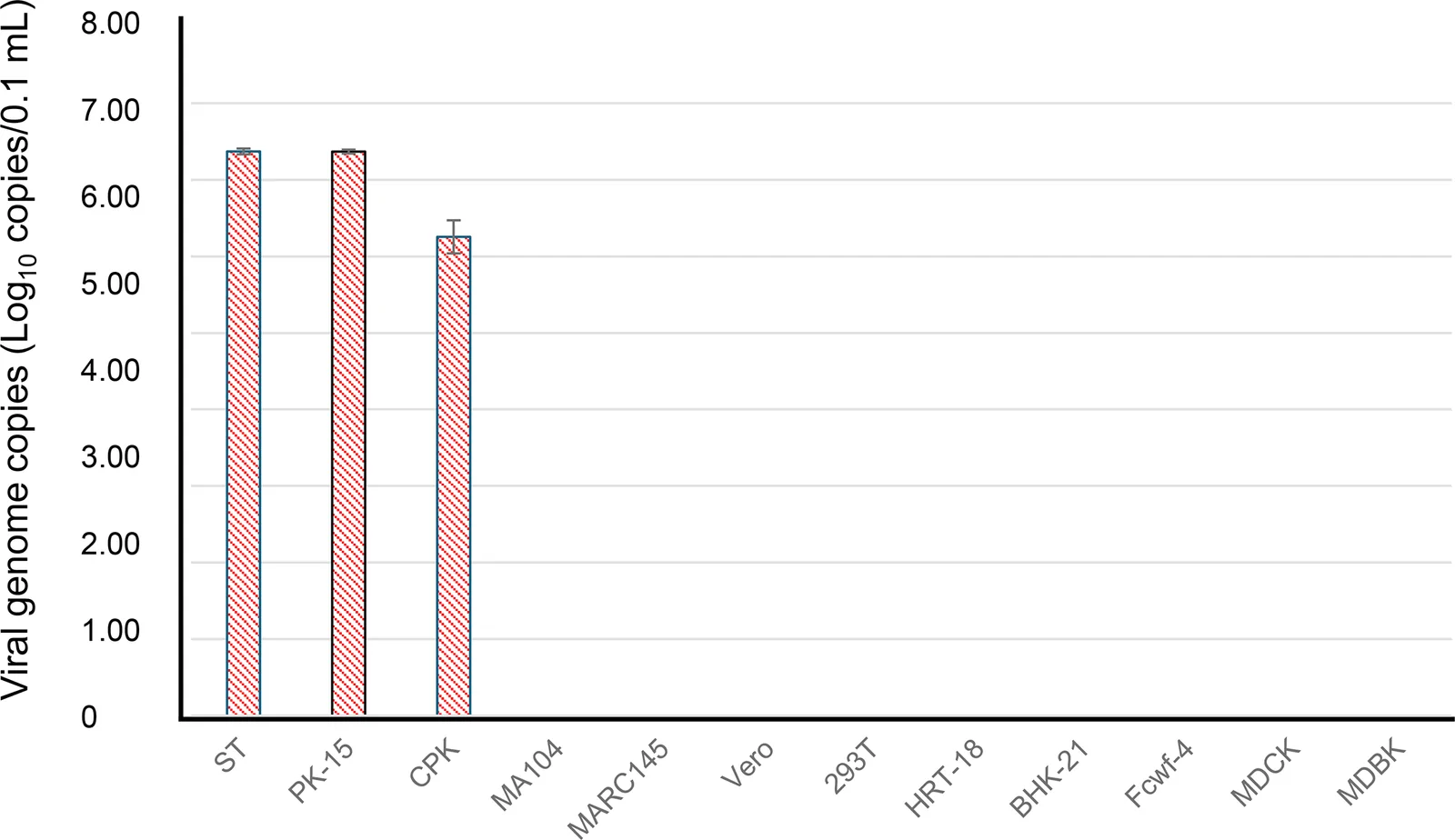
Viral genome copies of Ishi-6 in ST, PK15, CPK, MA104, MARC145, Vero, 293T, HRT-18, BHK-21, Fcwf-4, MDCK and MDBK cells at 4 dpi. Cells were infected with Ishi-6 at ~100 genomic copies per cell.

### Genome analyses of Ishi-6

The complete genome sequence of Ishi-6 was determined by deep sequencing, 5′ RACE and 3′-terminal amplification and sequencing. PCR products were successfully obtained by 5′ RACE, and a 677-nt 5′ UTR sequence was determined. The 5′ end of the obtained sequence was pyrimidine-rich; however, based on comparisons with the 5′-termini of other picornaviruses and the absence of a UU or UUU motif at the extreme 5′ end [17, 18], the obtained sequence may not represent the complete 5′ UTR. Secondary structure analysis predicted that Ishi-6 possesses a type II internal ribosome entry site (IRES) containing the core domains and conserved structural motifs, including a GNRA tetraloop motif and an A-bulge. These domains were followed by a pyrimidine tract and an AUG initiation codon in an optimal Kozak context. Comparison of this region with the corresponding sequence of the WOL showed 94.1% nt identity, and the H–L loop structures were highly similar (Fig. 4a).

**Fig. 4.**
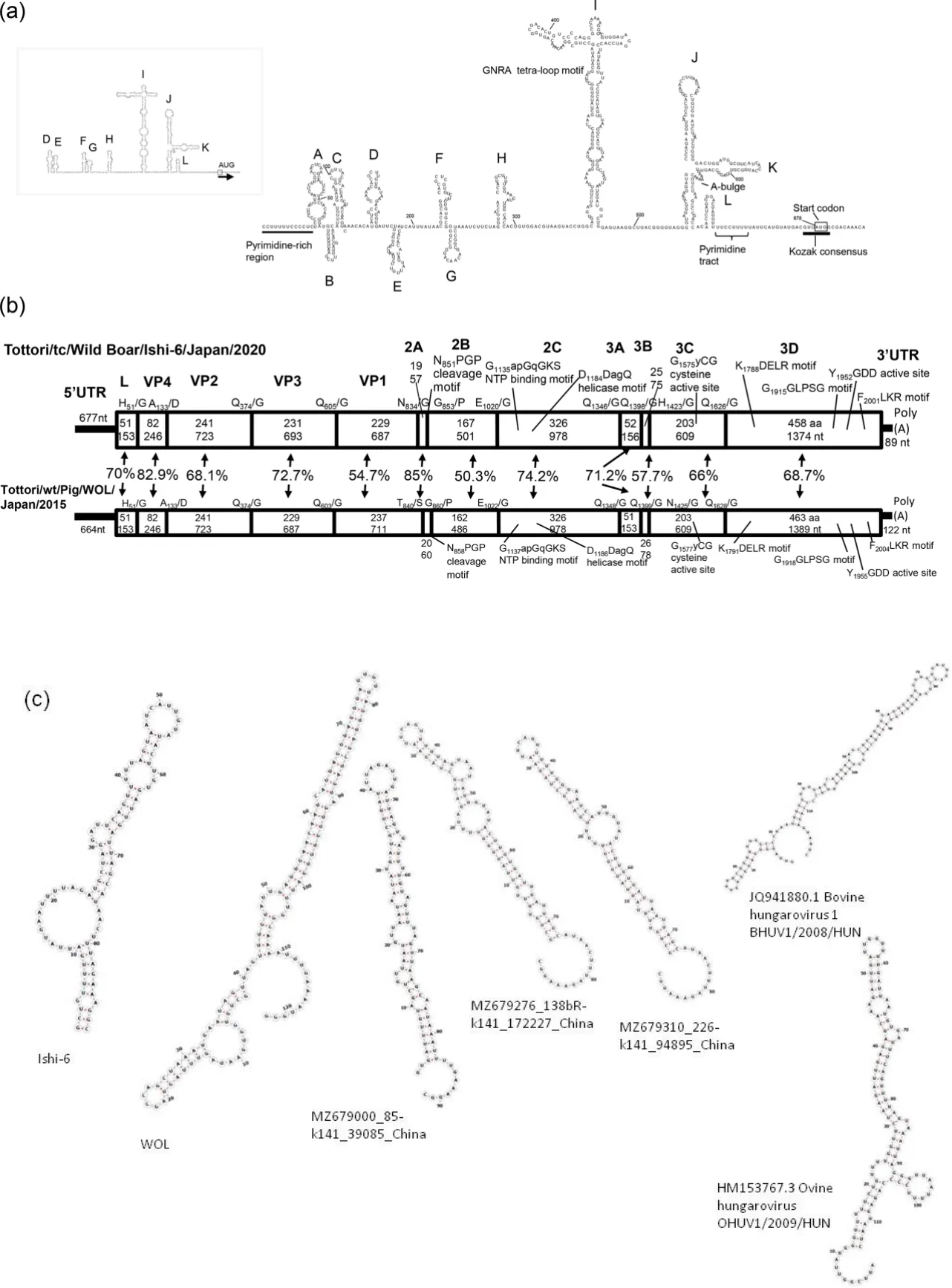
(**a**) Predicted secondary RNA structure of the Ishi-6 5′ UTR, including the IRES, generated using the RNAfold programme with reference to the TotV strain WOL (inset). The pyrimidine-rich region at the 5′-terminus, core IRES domains, conserved GNRA tetraloop and A-bulge motifs, pyrimidine tract at the 3′ end, Kozak consensus sequence and predicted polyprotein AUG start codon are indicated. (**b**) Schematic diagram of the Ishi-6 genome showing predicted cleavage sites, conserved picornavirus motifs and comparison with the genome structure of WOL. aa (upper numbers) and nt (lower numbers) lengths are indicated in each genomic region. aa identities (%) between Ishi-6 and WOL are shown for each genomic region. (**c**) Predicted secondary RNA structure of the 3′ UTR of Ishi-6, WOL, Chinese TotVs MZ679000, MZ679276 and MZ679310, and bovine and ovine hunniviruses (JQ941880 and HM153767).

Comparison of the coding regions between Ishi-6 and WOL revealed relatively high aa identities in the 2A and VP4 regions (85.0 and 82.9%, respectively), whereas the VP1 and 2B regions showed lower identities (54.7 and 50.3%, respectively). Both strains possessed a leader protein region and 2A protein containing an NPG↓P motif. The 2C, 3C and 3D proteins shared aa identities of 74.2, 66.0 and 68.7%, respectively. Nevertheless, conserved functional motifs were retained, including the GapGqGKS NTP-binding motif and DDagQ helicase motif in 2C, the GyCG cysteine active-site motif in 3C and the KDELR, GGLPSG, YGDD and FLKR motifs in 3D, all of which were conserved at corresponding positions (Fig. 4b).

The 3′ UTR of Ishi-6 comprised 89 nt, which was 33 nt shorter than that of WOL, and shared 42.6% nt identity with the corresponding region of WOL. Secondary structure prediction using RNAfold identified a single relatively large stem-loop structure in both strains. Similar structures were also observed in three other TotV strains from China with nearly complete 3′ UTR sequences, as well as in bovine and ovine hunniviruses (Fig. 4c).

### Phylogenetic analysis and pairwise sequence comparison

Complete aa sequences of the Ishi-6 polyprotein, P1 and 2C+3 CD regions were aligned with those of TotVs and related viruses, including malagasivirus and hunnivirus, retrieved from the GenBank/EMBL/DDBJ/PIR database. Pairwise sequence comparisons and phylogenetic analyses using the ML method were subsequently performed.

In pairwise comparisons of the polyprotein sequences, Ishi-6 shared 67.17–83.85% aa identity (64.33–77.35% nt identity) with other TotVs, whereas identities with malagasivirus and hunnivirus were markedly lower, at 34.87% (42.68%) and 33.89% (41.57%), respectively (Table 1). In the polyprotein phylogenetic tree, TotVs were divided into three distinct groups supported by bootstrap values of ≥96, and Ishi-6 was most closely related to the U.S. pig slurry-derived strain MW504594. The WOL strain, detected in Japanese pigs in 2015, clustered with Vietnamese porcine strains and the U.S. pig slurry-derived strain (Fig. 5a).

**Fig. 5.**
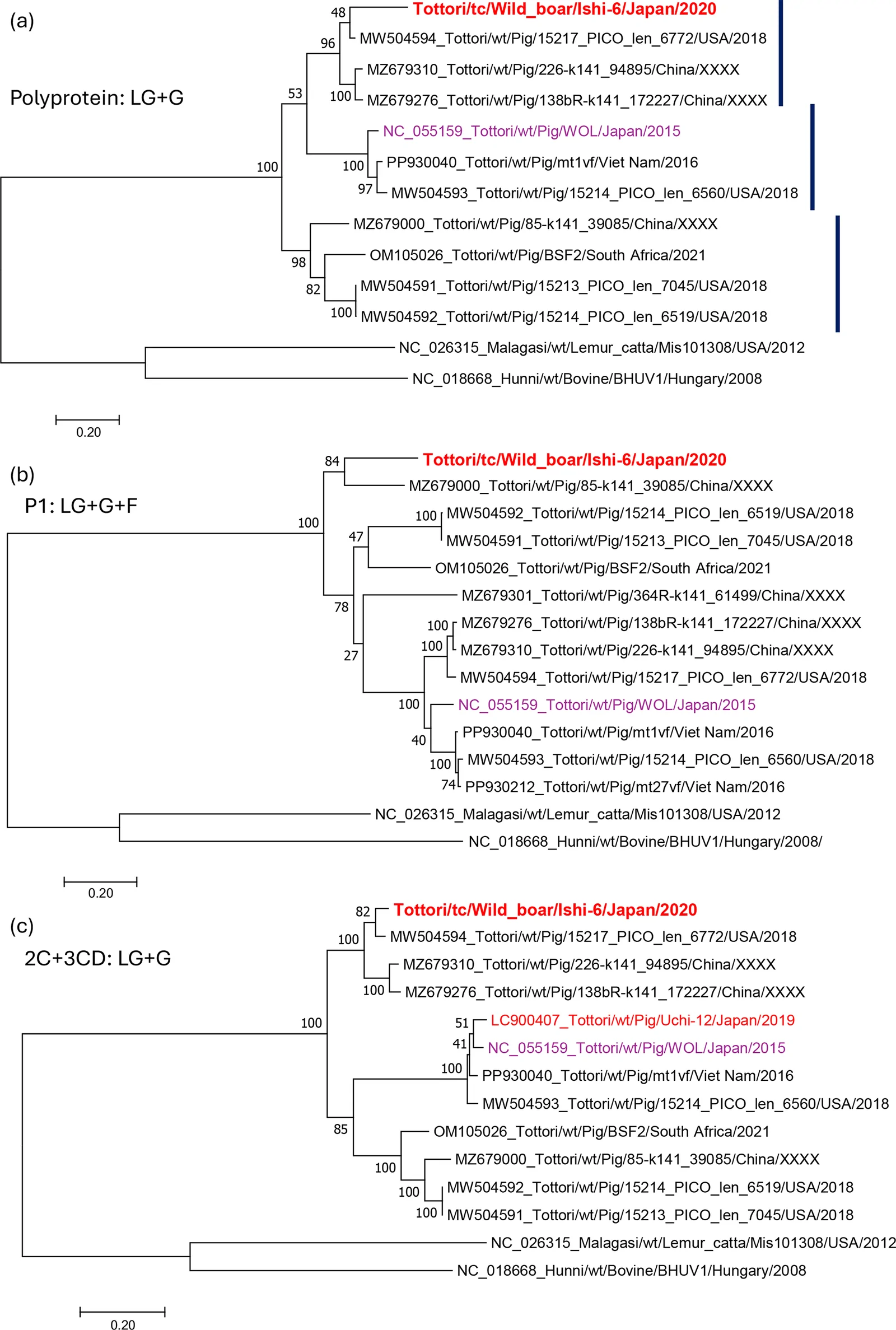
Phylogenetic analysis based on the aa sequences of the polyprotein (**a**), P1 (**b**) and 2C+3 CD (**c**) regions of TotV and related strains retrieved from the GenBank/EMBL/DDBJ/PIR databases. The phylogenetic trees were constructed using the ML method implemented in mega12 with the best-fit substitution models (LG+G for the polyprotein and 2C+3 CD trees, and LG+G+F for the P1 tree). Tree robustness was evaluated by bootstrap analysis with 1,000 replicates. The scale bar indicates the corrected genetic distance. The Japanese TotVs Ishi-6 and Uchi-12 are shown in red, with Ishi-6 highlighted in bold, whereas WOL is shown in purple.

**Table 1.** Pairwise aa (lower left) and nt (upper right) sequence identity (%) comparison of polyprotein of TotVs and related viruses. The values showing the highest identity to the Ishi-6 are highlighted in bold

	Ishi-6/2020	NC_055159	MW504591	MW504592	MW504593	MW504594	MZ679000	MZ679276	MZ679301	PP930040	OM105026	NC_026315	NC_018668
Tottori/tc/Wild Boar/Ishi-6/Japan/2020		64.33	64.92	64.90	64.73	**77.35**	67.30	73.69	73.37	64.57	65.74	42.68	41.57
NC_055159_Tottori/wt/Pig/WOL/Japan/2015	67.17		63.73	63.73	83.44	68.76	64.86	69.46	69.29	83.84	66.33	41.99	41.02
MW504591_Tottori/wt/Pig/15213_PICO_len_7045/USA/2018	70.42	67.38		99.97	64.73	65.54	72.80	66.08	65.98	64.56	74.09	42.63	41.10
MW504592_Tottori/wt/Pig/15214_PICO_len_6519/USA/2018	70.42	67.38	99.95		64.73	65.52	72.80	66.06	65.97	64.56	74.07	42.63	41.10
MW504593_Tottori/wt/Pig/15214_PICO_len_6560/USA/2018	67.79	91.59	68.05	68.05		68.22	64.46	68.67	68.43	86.76	65.89	42.17	41.15
MW504594_Tottori/wt/Pig/15217 _PICO_len_6772/USA/2018	**83.85**	73.88	70.74	70.74	73.88		66.27	80.11	80.72	68.81	67.30	43.24	41.86
MZ679000_Tottori/wt/Pig/85-k141 _39085/China/XXXX	73.17	68.06	80.93	80.88	68.06	71.80		65.89	65.97	64.72	70.60	42.01	41.47
MZ679276_Tottori/wt/Pig/138bR-k141 _172227/China/XXXX	78.02	74.26	70.87	70.82	74.02	89.57	70.93		89.50	69.22	66.27	42.51	41.85
MZ679301_Tottori/wt/Pig/364 R-k141_61499/China/XXXX	78.26	74.02	70.82	70.77	73.88	89.81	71.22	95.83		69.10	66.06	43.16	41.71
PP930040_Tottori/wt/Pig/mt1vf/Viet Nam/2016	67.70	92.97	67.57	67.57	95.60	73.93	68.02	74.31	74.36		65.51	42.07	41.04
OM105026_Tottori/wt/Pig/BSF2/South Africa/2021	69.85	69.38	82.19	82.19	69.43	71.46	78.14	71.11	70.96	69.10		42.35	41.33
NC_026315_Malagasi/wt/Lemur_catta/Mis101308/USA/2012	34.87	33.91	35.39	35.39	34.15	34.70	34.54	34.51	35.17	34.08	34.60		46.77
NC_018668_Hunni/wt/Bovine/BHUV1/Hungary/2008	33.89	33.75	33.64	33.64	33.75	33.71	34.24	33.62	33.89	33.89	33.92	40.26	

For the P1 region, Ishi-6 showed the highest aa identity (74.18%) and nt identity (67.93%) with the Chinese porcine strain MZ679000 (Table 2). The phylogenetic topology of the P1 region differed from that of the polyprotein tree, with Ishi-6 clustering together with strain MZ679000, consistent with the high sequence identity observed between the two strains (Fig. 5b).

**Table 2.** Pairwise aa (lower left) and nt (upper right) sequence identity (%) comparison of P1 of TotVs and related viruses. The values showing the highest identity to the Ishi-6 are highlighted in bold

	Ishi-6/2020	NC_055159	MW504591	MW504592	MW504593	MW504594	MZ679000	MZ679276	MZ679301	MZ679310	PP930040	PP930212	OM105026	NC_026315	NC_018668
Tottori/tc/Wild Boar/Ishi-6/Japan/2020		63.93	62.87	62.83	64.48	63.93	**67.93**	65.11	64.35	64.01	63.97	64.48	63.25	43.03	42.59
NC_055159_Tottori/wt/Pig/WOL/Japan/2015	66.88		63.71	63.67	77.06	73.84	65.70	76.58	64.51	75.78	77.40	76.81	67.43	44.36	43.51
MW504591_Tottori/wt/Pig/15213_PICO_len_7045/USA/2018	68.90	70.63		99.96	65.53	63.76	63.63	65.32	64.68	64.64	64.89	65.11	67.98	43.76	42.89
MW504592_Tottori/wt/Pig/15214_PICO_len_6519/USA/2018	68.90	70.63	99.87		65.49	63.71	63.59	65.27	64.64	64.60	64.85	65.06	67.94	43.76	42.89
MW504593_Tottori/wt/Pig/15214_PICO_len_6560/USA/2018	67.76	87.83	70.89	70.89		73.67	64.69	75.32	64.68	74.47	87.58	88.68	66.16	43.76	43.35
MW504594_Tottori/wt/Pig/15217_PICO_len_6772/USA/2018	66.88	86.08	70.63	70.63	85.44		64.94	82.57	64.56	83.42	74.14	74.05	67.00	44.39	43.78
MZ679000_Tottori/wt/Pig/85-k141_39085/China/XXXX	**74.18**	70.29	69.41	69.28	70.42	70.80		65.66	63.63	64.77	64.48	65.32	64.30	43.43	43.74
MZ679276_Tottori/wt/Pig/138bR-k141_172227/China/XXXX	66.50	86.33	70.13	70.00	86.08	95.30	71.05		65.15	91.50	75.53	75.95	66.84	44.75	43.74
MZ679301_Tottori/wt/Pig/364 R-k141_61499/China/XXXX	68.48	70.00	71.39	71.27	70.13	69.62	67.26	69.87		64.43	65.70	65.19	65.11	42.33	42.85
MZ679310_Tottori/wt/Pig/226-k141_94895/China/XXXX	66.62	86.58	70.00	69.87	86.08	95.43	71.05	98.60	70.13		75.40	75.53	66.29	44.55	43.78
PP930040_Tottori/wt/Pig/mt1vf/Viet Nam/2016	67.64	88.34	70.51	70.51	97.72	85.95	70.16	86.20	70.00	86.20		91.72	66.67	44.20	43.06
PP930212_Tottori/wt/Pig/mt27vf/Viet Nam/2016	67.76	87.96	71.01	71.01	98.35	86.08	70.54	86.33	70.13	86.33	98.35		66.71	43.76	43.22
OM105026_Tottori/wt/Pig/BSF2/South Africa/2021	67.38	73.42	74.62	74.62	72.41	72.78	70.51	72.03	71.77	72.15	72.28	72.28		44.40	43.26
NC_026315_Malagasi/wt/Lemur_catta/Mis101308/USA/2012	38.44	38.25	39.45	39.45	38.37	38.10	38.08	38.22	37.53	38.46	38.49	38.37	38.97		48.18
NC_018668_Hunni/wt/Bovine/BHUV1/Hungary/2008	36.54	35.60	37.70	37.70	36.09	36.56	38.15	36.31	36.22	36.93	36.09	36.59	37.45	42.23	

Analysis of the 2C+3 CD region showed that Ishi-6, similar to the polyprotein analysis, was most closely related to the U.S. strain MW504594, sharing 95.16% aa identity and 86.79% nt identity (Table 3). The phylogenetic tree based on the 2C+3 CD region showed a topology similar to that of the polyprotein tree, with Ishi-6 clustering together with strain MW504594 and forming a cluster with Chinese porcine strains. Tottori/wt/Pig/Uchi-12/Japan/2019, which was detected in a 4-month-old pig reared in Kanagawa Prefecture, was closely related to the WOL strain (Fig. 5c).

**Table 3.** Pairwise aa (lower left) and nt (upper right) sequence identity (%) comparison of 2C+3 CD of TotVs and related viruses. The values showing the highest identity to the Ishi-6 are highlighted in bold

	Ishi-6/2020	LC900407	NC_055159	MW504591	MW504592	MW504593	MW504594	MZ679000	MZ679276	MZ679310	PP930040	OM105026	NC_026315	NC_018668
Tottori/tc/Wild Boar/Ishi-6/Japan/2020		66.53	65.70	68.01	68.01	66.10	**86.79**	68.38	81.32	81.35	66.40	69.05	48.86	48.25
LC900407_Tottori/wt/Pig/Uchi-12/Japan/2019	70.11		87.46	65.63	65.60	87.60	67.20	65.46	65.60	65.40	87.96	66.73	47.30	48.09
NC_055159_Tottori/wt/Pig/WOL/Japan/2015	69.81	95.29		65.46	65.50	87.93	67.44	65.20	65.50	66.37	87.56	67.03	47.30	46.62
MW504591_Tottori/wt/Pig/15213_PICO_len_7045/USA/2018	75.40	70.01	69.81		99.97	65.36	68.51	80.07	68.35	68.68	66.17	77.49	48.40	47.1
MW504592_Tottori/wt/Pig/15214_PICO_len_6519/USA/2018	75.40	70.01	69.81	100.00		65.40	68.51	80.11	68.35	68.68	66.20	77.49	48.40	47.1
MW504593_Tottori/wt/Pig/15214_PICO_len_6560/USA/2018	70.21	94.78	95.09	70.41	70.41		66.53	65.40	65.13	66.17	87.26	66.60	47.76	46.98
MW504594_Tottori/wt/Pig/15217_PICO_len_6772/USA/2018	**95.16**	68.91	68.91	74.50	74.50	69.51		68.25	81.25	80.78	67.34	69.42	49.29	48.18
MZ679000_Tottori/wt/Pig/85-k141_39085/China/XXXX	75.71	70.41	70.31	90.93	90.93	70.21	75.30		66.90	67.78	66.10	74.93	47.57	47.03
MZ679276_Tottori/wt/Pig/138bR-k141_172227/China/XXXX	87.60	68.10	68.41	74.80	74.80	68.71	89.31	73.19		90.12	66.00	67.04	47.54	47.53
MZ679310_Tottori/wt/Pig/226-k141_94895/China/XXXX	88.51	69.01	69.11	74.90	74.90	69.41	89.42	73.49	95.77		66.67	67.67	48.99	47.03
PP930040_Tottori/wt/Pig/mt1vf/Viet Nam/2016	70.21	95.49	95.79	70.01	70.01	95.79	69.01	70.11	68.30	69.41		66.17	47.86	47.27
OM105026_Tottori/wt/Pig/BSF2/South Africa/2021	75.00	71.11	71.11	87.40	87.40	71.31	73.99	85.38	72.98	73.69	71.21		47.27	47.05
NC_026315_Malagasi/wt/Lemur_catta/Mis101308/USA/2012	40.63	39.03	39.03	40.34	40.34	39.23	40.44	40.04	40.24	41.13	39.23	39.35		53.58
NC_018668_Hunni/wt/Bovine/BHUV1/Hungary/2008	40.51	40.94	40.74	39.33	39.33	40.45	40.31	39.72	40.31	40.41	40.35	40.06	48.47	

### Recombination analysis

Phylogenetic analyses of the P1 region showed that Ishi-6 clustered with the Chinese porcine strain MZ679000, whereas in the 2C+3 CD region it clustered with the U.S. pig slurry-derived strain MW504594. This incongruence in phylogenetic topology suggested the occurrence of a possible homologous recombination event. To investigate this possibility, recombination analyses were performed using the sequences of Ishi-6, MZ679000 and MW504594 with SimPlot and RDP.

Similarity plot analysis revealed that Ishi-6 shared higher or comparable nt similarity with MZ679000 in the 5′ UTR to VP1 regions, whereas higher similarity to MW504594 than to MZ679000 was observed in the 2A–3′ UTR region. These findings supported the presence of a recombination event (Fig. 6b). Bootstrap scanning analysis implemented in RDP identified a putative recombination breakpoint near the VP1–2A junction (Fig. 6a, c). Ishi-6, MZ679000 and MW504594 were inferred to represent the recombinant, major parent and minor parent, respectively. This recombination event was strongly supported by multiple methods implemented in RDP, with *P*-values of 6.388×10^−35^ (RDP), 1.392×10^−7^ (GENECONV), 1.082×10^−33^ (BootScan), 3,000×10^−199^ (MaxChi), 4.061×10^−25^ (Chimaera), 2.988×10^−58^ (SiScan) and 3.334×10^−10^ (3Seq) (Fig. 6d).

**Fig. 6.**
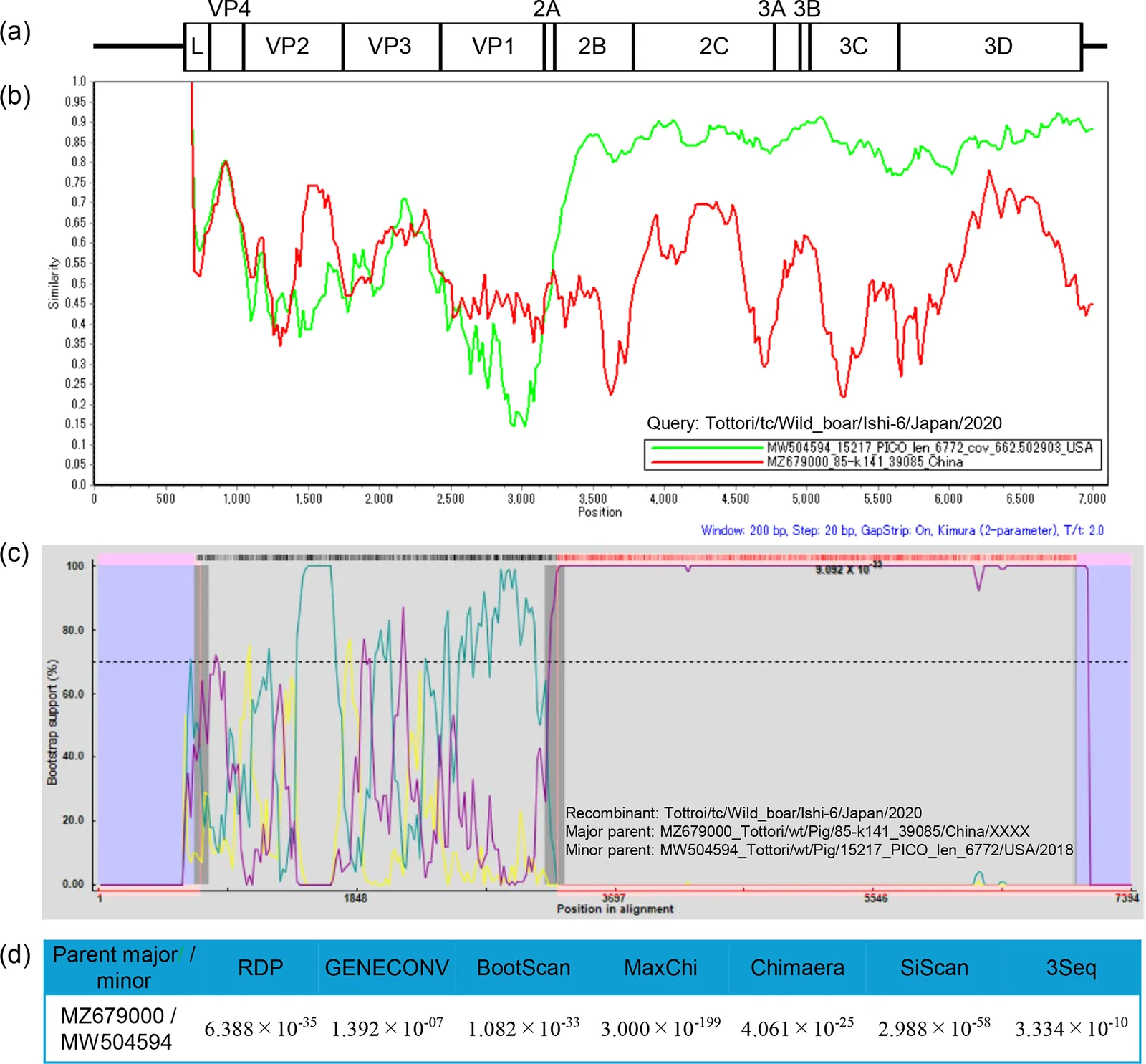
Genome organization and recombination analysis of the Ishi-6 strain. (**a**) Schematic representation of the Ishi-6 genome organization. (**b**) Similarity plots of the whole-genome sequences of MW504594 (green) and MZ679000 (red) using Ishi-6 as the query sequence. (**c**) BootScan recombination analysis for MZ679000 vs MW504594 (yellow), MZ679000 vs Ishi-6 (blue-green) and MW504594 vs Ishi-6 (purple). (**d**) *P*-values for each recombination detection method calculated using the RDP.

### LDA-based host prediction

LDA-based host prediction suggested that TotVs clustered within vertebrate with the 90% confidence interval (Fig. 7).

**Fig. 7.**
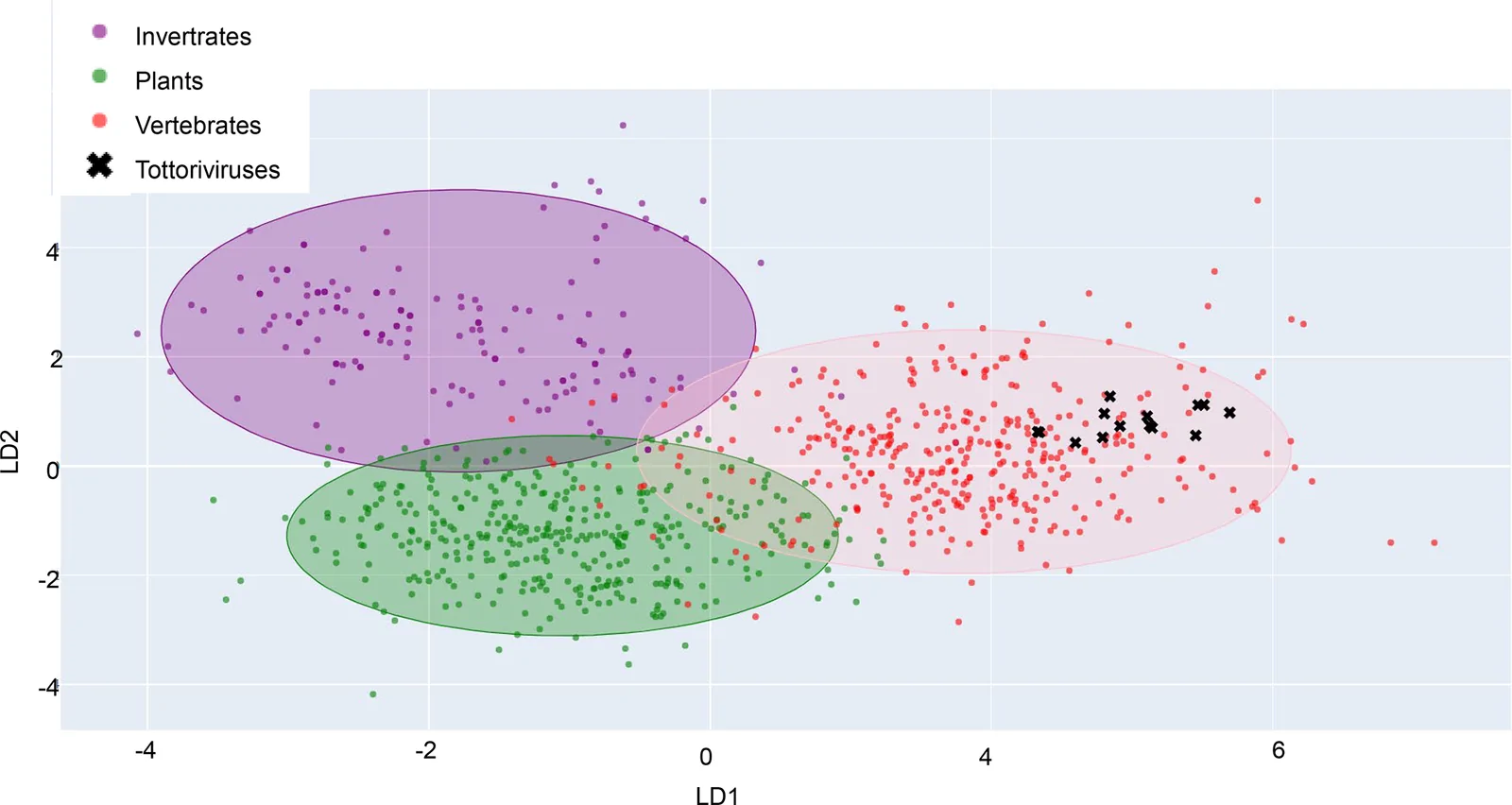
Canonical score plot from discriminant analysis of TotVs used to classify viral sequences into host groups based on four mononucleotide and 16 dinucleotide frequencies. The graph illustrates the separation of groups using the two most influential factors. Viruses infecting invertebrates are represented by purple circles, plant-infecting viruses by green circles and vertebrate-infecting viruses by red circles. The lines indicate the 90% confidence intervals.

### Prevalence investigation

A prevalence investigation of 280 healthy juvenile wild boars hunted in Ishikawa and Toyama Prefectures between 2018 and 2023 identified nine TotV-positive animals (3.2%), including the wild boar from which Ishi-6 was isolated. The Cq values of the positive samples ranged from 26.56 to 34.58, whereas the Ishi-6-positive sample exhibited a lower Cq value of 21.47. In contrast, all rectal swab samples collected from 70 pigs on 7 farms in Ishikawa Prefecture in 2021 tested negative for the virus (Table 4).

**Table 4.** Total number of wild boar and pig faecal samples and the number of samples positive in qRT-PCR targeting the 5′ UTR of TotV

		2017	2018	2019	2020	2021	2022	2023	Total
Wild boar	Sample number	–	35	52	23	34	66	70	**280**
	Samples positive in qRT-PCR	–	2	1	3	0	3	0	**9**
Pig	Sample number	–	–	–	–	70	–	–	**70**
	Samples positive in qRT-PCR	–	–	–	–	0	–	–	**0**

## Discussion

During an investigation of enteric viruses in wild boars using ST cell-based isolation followed by deep sequencing, a novel TotV was identified. As this represents the first reported isolation of a member of the genus *Tottorivirus*, comprehensive characterization of the virus was warranted. Accordingly, the present study investigated this virus through TEM observation, cell tropism analysis, genome characterization and comparative genetic analyses between the Ishi-6 strain and other TotV sequences available in the GenBank/EMBL/DDBJ/PIR database.

During the isolation of Ishi-6, inoculated ST cells developed characteristic CPE, including cell rounding, monolayer disruption and reticular degeneration by 3 dpi, consistent with cytopathic changes previously reported for picornavirus infections in swine-derived cell lines [19]. Ultrastructural analysis by TEM revealed quasicrystalline arrays of virus-like structures associated with cytoplasmic vesicles in infected cells, a feature commonly observed in picornavirus-infected cells and consistent with active viral replication [20].

Cell tropism analysis demonstrated that Ishi-6 replicated productively in three porcine-derived cell lines, ST, PK15 and CPK, but not in cell lines derived from other species, including primates (Vero, MA104, MARC-145 and 293T), bovines (MDBK), hamsters (BHK-21), dogs (MDCK), cats (Fcwf-4) or humans (HRT-18). This restricted host cell tropism is consistent with the detection of TotVs exclusively in swine and wild boars and suggests that TotVs may exhibit host-specific tropism for *Sus scrofa*. Although cellular receptors and other host factors are known to influence picornavirus tropism [21–23], the receptor(s) involved in TotV infection remain unknown and warrant further investigation. LDA-based host prediction placed TotVs within the vertebrate virus cluster, further supporting the adaptation of TotVs to *Sus scrofa* hosts.

Although CPE was observed in all three porcine-derived cell lines, it was relatively mild. RT-qPCR detected viral genome copies even in wells without visible CPE, and plaque formation was not observed in any of the tested cell lines. Ishi-6-infected cells were maintained in culture medium supplemented with TPCK-treated trypsin. Trypsin supplementation has been reported to facilitate the isolation of several viruses, including picornaviruses [24]. In particular, supplementation with trypsin has been reported to be required for the isolation of Enterovirus G (*Enterovirus geswini*), another member of the family *Picornaviridae* [25], although it may become dispensable after the third passage [26]. In the present study, viral replication was confirmed by RT-qPCR in the culture supernatant of ST cells maintained without trypsin supplementation (data not shown). Ishi-6 was successfully passaged up to the 16th passage; however, no visible CPE was observed in the absence of trypsin supplementation (data not shown), suggesting that although trypsin is not absolutely required for viral genome replication itself, unlike in Enterovirus G [26], it may be important for efficient cytopathic infection cycles. In this context, RT-qPCR proved essential for accurately monitoring viral replication.

Genome characterization of Ishi-6 confirmed its classification as a member of the genus *Tottorivirus* within the family *Picornaviridae*. The 5′ UTR contained a type II IRES with conserved structural elements, including a GNRA tetraloop motif and an A-bulge, followed by a pyrimidine tract and an AUG initiation codon in an optimal Kozak context, consistent with features previously described for WOL [4]. The coding region encoded both a leader protein and a 2A protein containing an NPG↓P motif, characteristic genomic features of this genus. The 3′ UTR is known to play important roles in viral RNA replication and translation in picornaviruses [27, 28]. The 3′ UTRs of TotVs are relatively short, ranging from 89 to 122 nt. Although the nt sequence identity between the 3′ UTRs of Ishi-6 and WOL was relatively low (42.6%), RNA secondary structure prediction identified a relatively large stem-loop structure conserved among all the analysed TotV strains. This finding suggests that conservation of RNA secondary structure, rather than the primary nt sequence, may be important for the replication or translation of TotVs.

Among the available TotV strains in the GenBank/EMBL/DDBJ/PIR database, Ishi-6 exhibited the highest polyprotein aa identity (83.85%) with the U.S. pig slurry-derived strain MW504594, whereas the lowest identity (67.17%) was observed with the Japanese strain WOL (Table 1). Similarly, within the P1 region, Ishi-6 showed the highest identity with the Chinese porcine strain MZ679000, whereas identity with WOL remained relatively low at 66.88% (Table 2). In contrast, aa identity within the 2C+3 CD region between Ishi-6 and the U.S. pig slurry-derived strain MW504594 was notably high (95.16%), raising the possibility that closely related TotVs may circulate across geographically distant regions. Furthermore, the lowest aa identity observed within the P1 region was 66.5% (between Ishi-6 and MZ679276). In the VP1 region, the lowest identity was 52.32% between Ishi-6 and the Viet Nam strain PP930040 (Tables 2 and S2). These findings indicate substantial genetic diversity within the capsid region of TotVs. Phylogenetic analysis based on the P1 region did not reveal distinct clustering patterns, precluding a robust genotype classification at present; however, additional sequence data may enable more robust classification in the future.

Phylogenetic analysis revealed incongruence between the P1 region, in which Ishi-6 clustered with the Chinese strain MZ679000, and the 2C+3 CD region, in which it grouped with the U.S. strain MW504594. This discordance strongly suggested a homologous recombination event. Similarity plot and RDP-based recombination analyses further supported this hypothesis, identifying a recombination breakpoint near the VP1–2A junction. These analyses suggested that Ishi-6 is a recombinant virus derived from an MZ679000-like strain (major parent) and an MW504594-like strain (minor parent), with strong statistical support (*P*<10⁻⁷ across all seven RDP methods). The P1/2A junction has been recognized as a recombination hotspot in picornaviruses [29, 30]. Acquisition of a genetically distinct P1 region may influence viral antigenicity, receptor usage or host range [31, 32], whereas conservation of the non-structural protein regions may reflect functional constraints on viral replication [33]. These findings suggest that recombination contributes to the genetic diversification of TotVs. Although the putative parental strains were identified from China and the USA, the evolutionary and epidemiological processes underlying the emergence of this recombinant virus in Japanese wild boars remain unclear and require further investigation.

A prevalence survey of 280 healthy juvenile wild boars in Ishikawa and Toyama Prefectures detected TotV RNA in nine individuals (3.2%) between 2018 and 2022, thereby confirming the presence of the virus in wild boars in this region, although the detection rate was relatively low. In contrast, all samples collected from pigs at a pig farm in Ishikawa Prefecture in 2021 tested negative for the virus. One possible explanation for the difference in detection results is the reinforcement of biosecurity measures on pig farms following the re-emergence of classical swine fever in Japan in 2018 [34]. These findings may suggest that the circulation dynamics of TotV differ between farm pigs and wild boar populations. However, the transmission routes and epidemiological relationships between domestic pigs and wild boars remain unclear.

In summary, we isolated and characterized a novel TotV, Ishi-6, from a wild boar in Japan. Ishi-6 exhibited productive replication primarily in porcine-derived cell lines and possessed genomic features characteristic of members of the genus *Tottorivirus*, including a type II IRES and an NPG↓P-containing 2A protein. Comparative genomic and phylogenetic analyses revealed marked genetic diversity among TotVs and identified Ishi-6 as a recombinant virus with distinct evolutionary origins for the capsid and non-structural regions. These findings support the role of recombination in the evolution and diversification of TotVs. In addition, detection of viral RNA in wild boars from Ishikawa and Toyama Prefectures indicates that TotVs circulate in wild boar populations in Japan. Collectively, this study expands current knowledge of the molecular virology, evolution and epidemiology of the genus *Tottorivirus*.

## Supplementary material

10.1099/jgv.0.002334 Supplementary Material 1.

## Abbreviations

BHK-21: Baby Hamster Syrian Kidney-21
CPE: cytopathic effect
CPK: Cloned Porcine Kidney
dpi: days post-infection
E-MEM: Eagle’s minimal essential medium
IRES: internal ribosome entry site
LDA: Linear Discriminant Analysis
MDBK: Madin–Darby Bovine Kidney
MDCK: Madin–Darby Canine Kidney
ML: maximum-likelihood
PK-15: Porcine Kidney-15
RACE: rapid amplification of cDNA ends
RDP: Recombination Detection Program
RT-PCR: reverse transcription polymerase chain reaction
RT-qPCR: real-time reverse transcription PCR
ST: swine testis
TEM: transmission electron microscopy
TotV: tottorivirus
TPCK: L-1-tosylamide-2-phenylmethyl chloromethyl ketone
